# Synthesis and crystal structure of topiramate azido­sulfate at 90 K and 298 K

**DOI:** 10.1107/S2056989022008799

**Published:** 2022-09-08

**Authors:** Prabhakar Priyanka, Bidarur K. Jayanna, Haruvegowda Kiran Kumar, Vinaya, Thayamma R. Divakara, Hemmige S. Yathirajan, Christopher Glidewell, Sean Parkin

**Affiliations:** a Department of Chemistry, B. N. M. Institute of Technology, Bengaluru-560 070, India; b Department of Studies in Chemistry, University of Mysore, Manasagangotri, Mysuru-570 006, India; c T. John Institute of Technology, Bengaluru-560 083, India; d School of Chemistry, University of St Andrews, St Andrews, Fife KY16 9ST, UK; e Department of Chemistry, University of Kentucky, Lexington, KY, 40506-0055, USA; Katholieke Universiteit Leuven, Belgium

**Keywords:** topiramate azido­sulfate, inter­mediate, anti-convulsant, crystal structure, absolute configuration

## Abstract

The low (90 K) and room (298 K) temperature crystal structures of topiramate azido­sulfate [systematic name 2,3:4,5-bis-*O*-(1-methyl­ethyl­idene)-β-d-fructo­pyran­ose azido­sulfate], C_12_H_19_N_3_O_8_S, an inter­mediate in the synthesis of the anti-convulsant drug topiramate, are described. Topiramate azido­sulfate (**I**) finds use as a reference impurity standard for topiramate. A modified synthesis and some spectroscopic details are also presented.

## Abstract 2

The synthesis, crystal structure, absolute configuration, spectroscopic and spectrometric details of topiramate azido­sulfate, a precursor to the anti-convulsant drug topiramate, are presented.

## 1. Chemical context

Topiramate, sold under the brand name Topamax (amongst others), is a carbonic anhydrase inhibitor, used alone or with other medications, to treat epilepsy and to prevent migraines (Maryanoff *et al.*, 1987 ▸; 1998 ▸; Maryanoff, 2009 ▸). It is also prescribed for the treatment of bipolar disorder, post-traumatic stress disorder, mood instability disorder, binge-eating disorders, bulimia nervosa and obesity (Silberstein *et al.*, 2005 ▸). The vibrational and thermal properties of topiramate were investigated by Sena *et al.* (2008 ▸). Topiramate azido­sulfate (a topiramate inter­mediate) is useful as a reference impurity standard. In view of the importance of topiramate and its derivatives, this paper reports the synthesis, crystal structure, and some spectroscopic data for topiramate azido­sulfate, C_12_H_19_N_3_O_8_S, at low and room temperature (90 K and 298 K). 
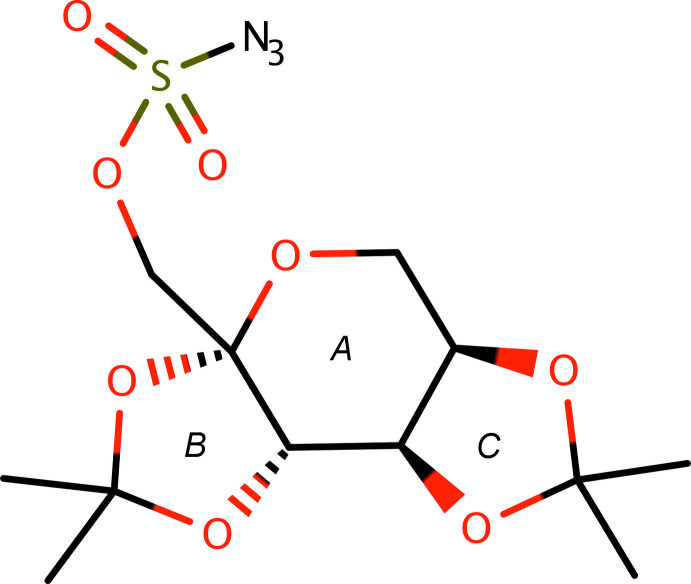


## 2. Structural commentary

**Figure 1:**
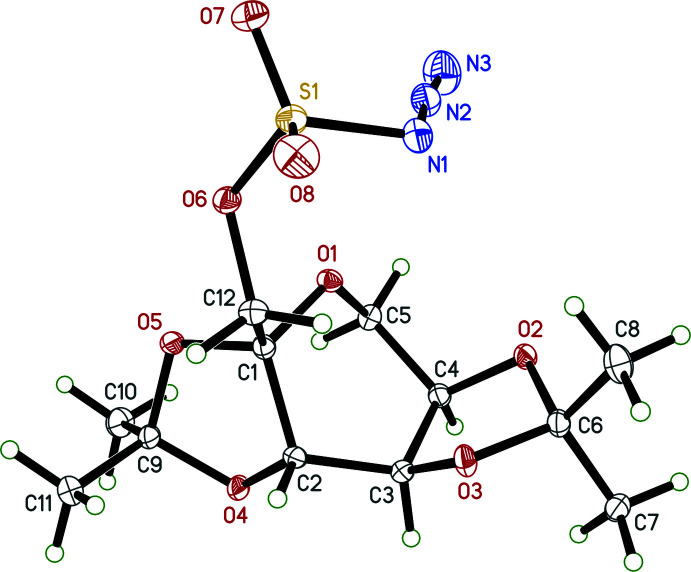
An ellipsoid plot of **I** (50% probability) for the structure at 90 K. The structure at 298 K is essentially unchanged, other than having much larger ellipsoids.

The mol­ecule of **I** (see scheme and Fig. 1 ▸) has a central core consisting of three fused rings: a pyran ring (labelled *A* in the scheme) with two fused dioxolane rings (labelled *B* and *C*). The points of fusion, atoms C1, C2, C3, C4 (Fig. 1 ▸), are contiguous chiral centres, the absolute configurations of which were confirmed unambiguously from the anomalous scattering by the sulfur to be 1*S*, 2*S*, 3*R*, 4*R* (see Flack, 1983 ▸; Hooft *et al.*, 2008 ▸; Parsons *et al.*, 2013 ▸). All three rings are non-planar, as indicated by their r.m.s. deviations from planarity (pyran *A*: 0.2597 Å; dioxolanes *B*, *C*: 0.1375, 0.1583 Å respectively) and by their Cremer–Pople (1975 ▸) ring-puckering parameters (Table 1 ▸). The distal carbon atoms of the dioxolane rings (*i.e.*, C6 and C9) each bear two methyl groups. The azido­sulfonate group attaches to atom C1 *via* a methyl­ene linker, with the position of the azide relative to the fused-ring system determined by torsions about four bonds (C1—C12, C12—O6, O6—S1, S1—N1), as summarized in Table 2 ▸. The structure was refined against both low-temperature (90 K) and room-temperature (298 K) data in order to analyse the behaviour of methyl atom C7 (see Section 3: *Supra­molecular features*). As there are no substantive differences, unless stated otherwise, numerical qu­anti­ties quoted in the discussion pertain to the low-temperature structure.

## 3. Supra­molecular features

**Figure 2:**
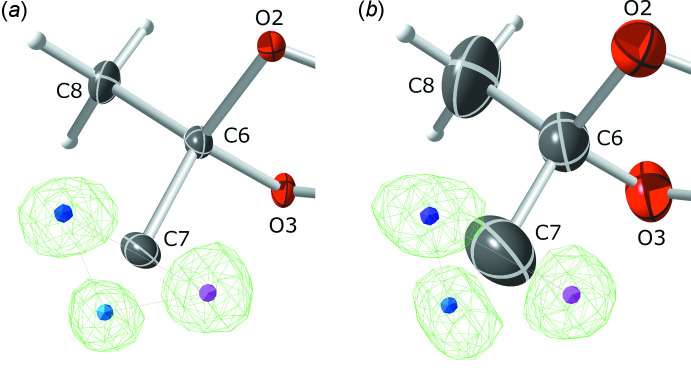
Difference-electron density showing the presence of well-ordered hydrogen atoms at both (*a*) 90 K and (*b*) 298 K for the methyl group at C7. Ellipsoids are drawn at the 50% probability level. Diagram generated using *ShelXle* (Hübschle *et al.*, 2011 ▸).

**Figure 3:**
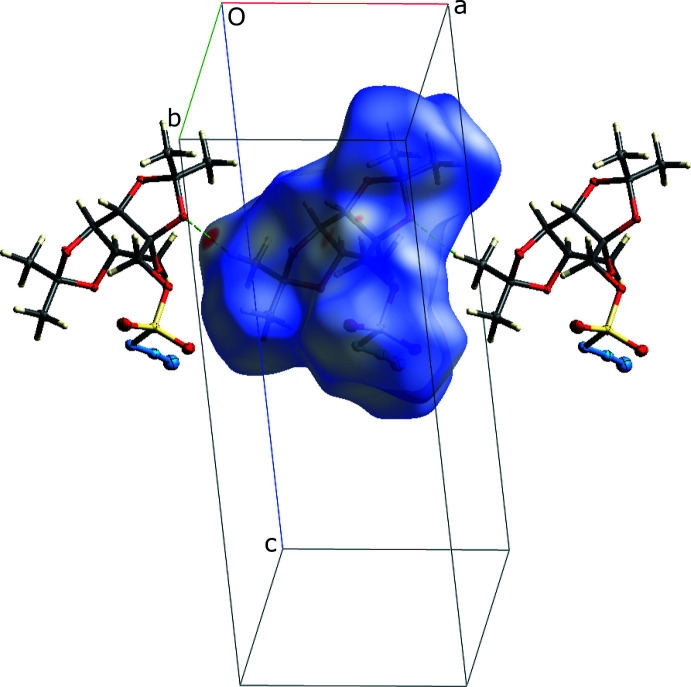
A plot of the Hirshfeld surface calculated over *d*_norm_ for **I** at 90 K, showing two adjacent mol­ecules. Hydrogen bonds are drawn as green dashed lines. The red spot at the left corresponds to the C7—H7*A*⋯O5^i^ [symmetry code: (i) *x* − 1, *y*, *z*] hydrogen bond (Table 3 ▸). The symmetry-equivalent red spot on the right side of the Hirshfeld surface is obscured from view.

**Figure 4:**
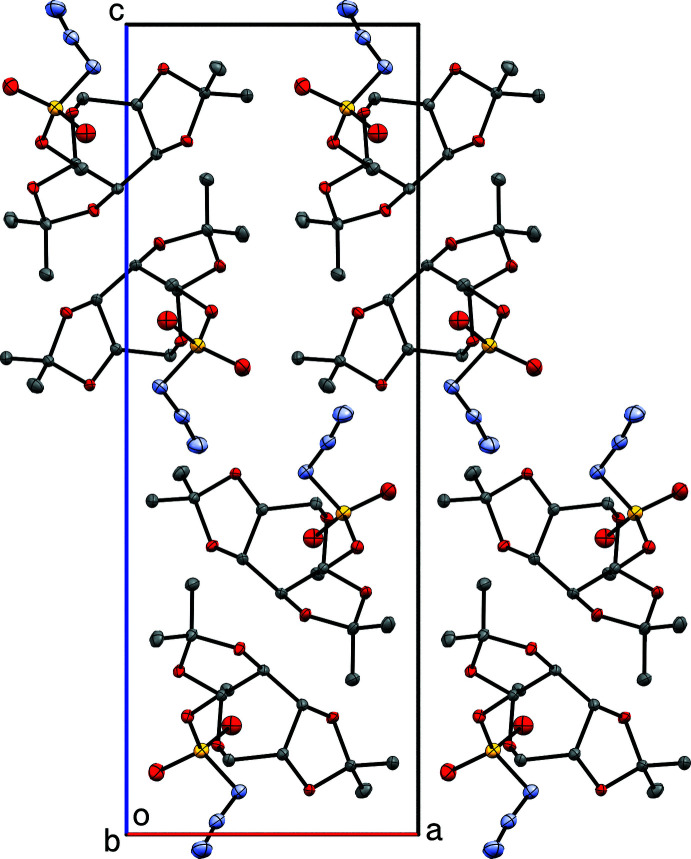
A packing plot of **I** viewed in projection down the *b*-axis, showing segregation of like groups, leading to the formation of double layers parallel to the *ab* plane. Diagram generated using *Mercury* (Macrae *et al.*, 2020 ▸).

**Figure 5:**
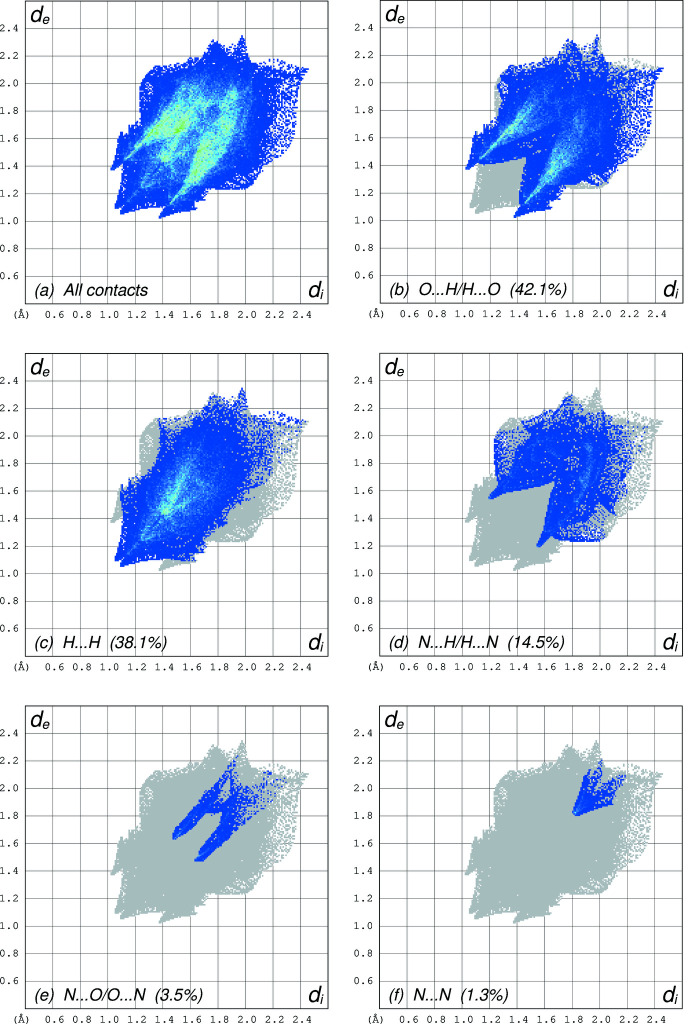
Fingerprint plots obtained from a Hirshfeld surface analysis for **I** at 90 K using *CrystalExplorer* (Spackman *et al.*, 2021 ▸). (*a*) All contacts, (*b*) O⋯H/H⋯O (42.1% coverage), (*c*) H⋯H (38.1%), (*d*) N⋯H/H⋯N (14.5%), (*e*) N⋯O/O⋯N (3.5%), (*f*) N⋯N (1.3%). All other contacts are negligible.

There are no strong inter­molecular inter­actions in crystals of **I**. The ‘HTAB’ instruction in *SHELXL* flags four ‘potential hydrogen bonds’ (Table 3 ▸), but two of these have very small C—H⋯O angles, such that the associated inter­action energy would be negligible (Wood *et al.*, 2009 ▸). The remaining two involve contacts between the methyl group at C7 with O1^i^ and O5^i^ of an adjacent mol­ecule [symmetry code: (i) *x* − 1, *y*, *z*], the latter being the stronger of the two. During structure analysis, the question arose of whether these contacts would be structurally significant, owing to the possibility of rapid methyl-group rotation at room temperature (Riddell & Rogerson, 1996 ▸; 1997 ▸). To answer this, the structure was also refined using room-temperature data. At low temperature (90 K) and room temperature (298 K), difference electron density for the three C7 methyl hydrogen atoms is very well resolved (Fig. 2 ▸), implying the absence of any disorder, rotational or static. Analysis of the Hirshfeld surface (Spackman & Jayatilaka, 2009 ▸) mapped over *d*_norm_ for **I** using *CrystalExplorer* (Spackman *et al.*, 2021 ▸) reveals only two (equivalent) prominent red spots, corresponding to the C7—H7*A*⋯O5^i^ inter­actions, in which the methyl group at C7 juts into a concave recess of an adjacent mol­ecule. These hydrogen bonds link the mol­ecules into chains that extend along the *a*-axis direction (Fig. 3 ▸). There are no especially short contacts involving the azido group; N2 and N3 are 3.118 (2) and 3.166 (2) Å, respectively from a screw-related sulfonyl O7 (*via* 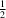 + *x*, 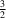 − *y*, 1 − *z*), but these are marginally greater than the sum of van der Waals radii of Bondi (1964 ▸). In spite of the lack of extensive inter­molecular inter­actions, the overall packing exhibits segregation of like groups, leading to double layers that extend in the *ab* plane (Fig. 4 ▸). A summary of the various atom–atom contacts obtained using *CrystalExplorer* fingerprint plots is given in Fig. 5 ▸.

## 4. Database survey

A search of the Cambridge Structural Database (version 5.43 with updates through June 2022; Groom *et al.*, 2016 ▸) for the three-ring core of topiramate plus the four methyl groups, but disregarding stereochemistry yielded 239 hits. A search fragment also including –CH_2_—*Z* (where *Z* is not H) attached to the equivalent of C1 in **I** returned 26 hits (21 excluding duplicates). A search using the keyword ‘topiramate’ gave only three hits, all being the structure of topiramate itself (with NH_2_ in place of N_3_ in **I**): SEQKAA (Maryanoff *et al.*, 1998 ▸) and duplicates SEQKAA01 (Kubicki *et al.*, 1999 ▸) and SEQKAA02 (Bolte, 2005 ▸). An amido derivative (with NHCHMePh in place of N_3_) is present as entry ZARCEC (Xie *et al.*, 2012 ▸). These crystal structures all have the symmetry of *P*2_1_2_1_2_1_, but pack differently from **I**. SEQKAA (and duplicates) form a tri-periodic hydrogen-bonded supra­molecular assembly, while ZARCEC forms *C*(4) chains (notation after Etter *et al.*, 1990 ▸).

## 5. Synthesis, crystallization and spectroscopic details

**Figure 6:**
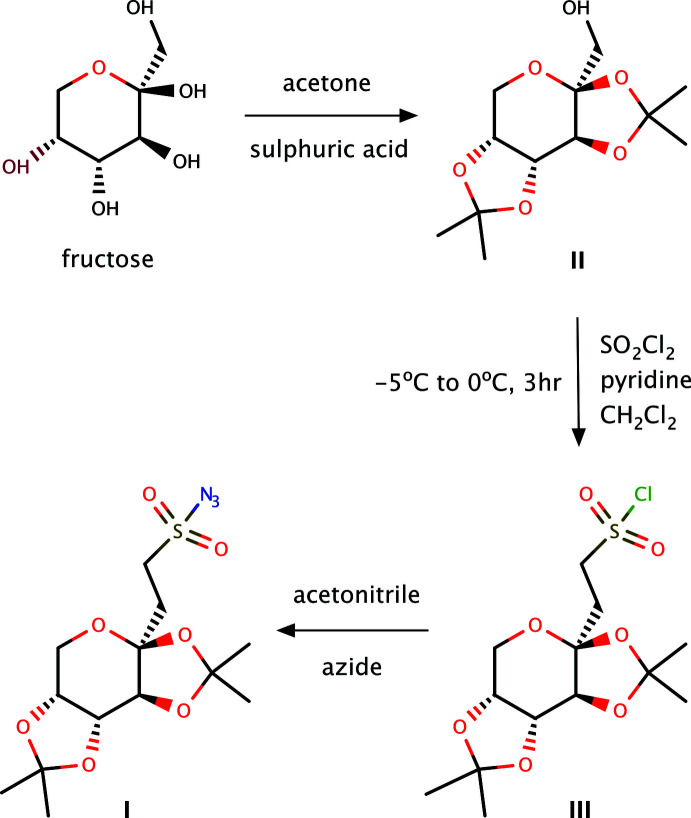
The reaction scheme for the synthesis of **I** starting from fructose.

Topiramate azido­sulfate was synthesized using a modification of procedures found in the literature (Maryanoff *et al.*, 1987 ▸; Kankan *et al.*, 2004 ▸; Arvai *et al.*, 2006 ▸; Koruyucu *et al.*, 2016 ▸). The synthesis involved three steps, *viz.*, (1) synthesis of 2,3:4,5 bis-*O*-(1-methyl­ethyl­idene)-β-d-fructo­pyran­ose, (2) synthesis of 2,3:4,5-bis-*O*-(1-methyl­ethyl­idene)-1-chloro­sulfate-β-d-fructo­pyran­ose, and (3) synthesis of topiramate azido­sulfate (**I**), as depicted in Fig. 6 ▸. X-ray quality crystals of **I** were obtained by crystallization from di­chloro­methane (m.p.: 358–359 K). Some spectroscopic details are as follows:

IR (cm^−1^): 2157 (N=N=N stretching); 1392 (S=O stretching); 1167 and 1081 (C—O stretching); ^1^H NMR: CDCl_3_ (400 MHz, δ ppm): 1.355 (3H, *s*, –CH_3_); 1.422 (3H, *s*, –CH_3_); 1.489 (3H, *s*, –CH_3_); 1.566 (3H, *s*, –CH_3_); 3.783–3.817 and 3.908–3.945 (2H, *dd*, –CH_2_); 4.246–4.268 (1H, *dd*, –CH); 4.306–4.332 (2H, *m*, –CH_2_); 4.398–4.424 (1H, *dd*, –CH); 4.622–4.649 (1H, *dd*, –CH). MS *m*/*z*: 364.03 (*M*—H)^+^

## 6. Refinement

Crystal data, data collection, and structure refinement details are summarized in Table 4 ▸. All H atoms were found in difference-Fourier maps, but subsequently included in the refinement using riding models, with constrained distances set to 0.98 Å (*R*CH_3_), 0.99 Å (*R*_2_CH_2_) and 1.00 Å (*R*_3_CH). *U*_iso_(H) parameters were set to values of either 1.2*U*_eq_ or 1.5*U*_eq_ (*R*CH_3_ only) of the attached atom. The absolute configuration was determined unambiguously from the anomalous scattering by sulfur using established methods (Flack, 1983 ▸; Hooft *et al.*, 2008 ▸; Parsons *et al.*, 2013 ▸).

## Supplementary Material

Crystal structure: contains datablock(s) global, I-90K, I-298K. DOI: 10.1107/S2056989022008799/vm2271sup1.cif

CCDC references: 2204998, 2204997

Additional supporting information:  crystallographic information; 3D view; checkCIF report

## Figures and Tables

**Table 1:** Cremer–Pople ring-puckering parameters (Å, °) for **I** at 90 K

Pyran	*Q*	θ	φ
*A*: O1, C1, C2, C3, C4, C5	0.6368 (16)	100.85 (14)	142.37 (15)
Dioxolane	*Q*_2_	φ_2_	
*B*: O4, C2, C1, O5, C9	0.3076 (15)	4.5 (3)	
*C*: O2, C4, C3, O3, C6	0.3539 (16)	133.4 (3)	

Cremer–Pople ring-puckering parameters were calculated using *PLATON* (Spek, 2020 ▸). For six-membered rings, the θ angles for ideal ‘boat’, ‘twist-boat’, and ‘screw-boat’ configurations are θ = 90° (boat, twist-boat) and θ = 112.5° (screw-boat). The φ values, are qu­anti­fied as either (60*k*)° (boat) or (60*k* + 30)° (twist-boat, screw-boat), with the one having *k* closest to an integer giving the conformation (Boeyens, 1978 ▸). Thus, pyran ring *A* in **I** is between ‘twist-boat’ and ‘screw boat’, though marginally closer to the former. For five-membered rings, φ qu­anti­fied as either (36*k*)° (‘envelope’) or (36*k* + 18)° (‘half-chair’) with *k* closest to an integer (Cremer & Pople, 1975 ▸), assigns dioxolane *B* as an ‘envelope’ configuration and dioxolane C as between ‘envelope’ and ‘half-chair’ conformations, though somewhat closer to the latter.

**Table 2:** Selected torsion angles (°) for **I** at 90 K

N1—S1—O6—C12	61.39 (12)	S1—O6—C12—C1	−133.17 (11)
O6—S1—N1—N2	71.03 (13)	C2—C1—C12—O6	177.58 (12)

**Table 3:** Hydrogen bonds and short inter­molecular contacts (Å, °) for **I** at 90 K

*D*—H⋯*A*	*D*—H	H⋯*A*	*D*⋯*A*	*D*—H⋯*A*
C7—H7*A*⋯O5^i^	0.98	2.50	3.456 (2)	164.8
C7—H7*A*⋯O1^i^	0.98	2.65	3.473 (2)	141.3
C5—H5*B*⋯O8^ii^	0.99	2.65	3.328 (2)	125.5
C12—H12*A*⋯O4^iii^	0.99	2.58	3.163 (2)	117.4

Symmetry codes: (i) *x* − 1, *y*, *z*; (ii) *x*, *y* − 1, *z*; (iii) −*x* + 1, *y* + 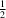 , −*z* + 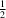 .

**Table 4:** Experimental details

	**I** at 90 K	**I** at 298 K
Crystal data		
Chemical formula	C_12_H_19_N_3_O_8_S	C_12_H_19_N_3_O_8_S
*M*_r_	365.36	365.36
Crystal system, space group	Orthorhombic, *P*2_1_2_1_2_1_	Orthorhombic, *P*2_1_2_1_2_1_
Temperature (K)	90	298
*a*, *b*, *c* (Å)	7.9857 (4), 9.0145 (4), 22.1621 (10)	8.0717 (8), 9.1135 (12), 22.506 (3)
*V* (Å^3^)	1595.39 (13)	1655.6 (3)
*Z*	4	4
Radiation type	Mo *K*α	Mo *K*α
μ (mm^−1^)	0.25	0.24
Crystal size (mm)	0.30 × 0.28 × 0.20	0.24 × 0.22 × 0.14

Data collection		
Diffractometer	Bruker D8 Venture dual source	Bruker D8 Venture dual source
Absorption correction	Multi-scan *SADABS* (Krause *et al.*, 2015 ▸)	Multi-scan (*SADABS*; Krause *et al.*, 2015 ▸)
*T*_min_, *T*_max_	0.845, 0.958	0.815, 0.959
No. of measured, independent and observed [*I* > 2σ(*I*)] reflections	22942, 3662, 3599	22913, 3786, 3523
*R*_int_	0.031	0.067
(sin θ/λ)_max_ (Å^−1^)	0.649	0.650

Refinement		
*R*[*F* ^2^ > 2σ(*F* ^2^)], *wR*(*F* ^2^), *S*	0.023, 0.060, 1.07	0.036, 0.099, 1.04
No. of reflections	3662	3786
No. of parameters	221	221
H-atom treatment	H-atom parameters constrained	H-atom parameters constrained
Δρ_max_, Δρ_min_ (e Å^−3^)	0.27, −0.27	0.20, −0.27
Absolute structure	Flack *x* determined using 1497 quotients [(*I* ^+^)−(*I* ^−^)]/[(*I* ^+^)+(*I* ^−^)] (Parsons *et al.*, 2013 ▸)	Flack *x* determined using 1388 quotients [(*I* ^+^)−(*I* ^−^)]/[(*I* ^+^)+(*I* ^−^)] (Parsons *et al.*, 2013 ▸)
Absolute structure parameter	−0.006 (18)	0.07 (5)

Computer programs: *APEX3* (Bruker, 2016 ▸), *SHELXT* (Sheldrick, 2015*a* ▸), *SHELXL2019/2* (Sheldrick, 2015*b* ▸), *XP* in *SHELXTL* (Sheldrick, 2008 ▸), *SHELXTL* (Sheldrick, 2008 ▸) and *publCIF* (Westrip, 2010 ▸).

## supplementary crystallographic information

### 2,3:4,5-Bis-O-(1-methylethylidene)-β-D-fructopyranose azidosulfate (I-90K) Crystal data

**Table:**

C_12_H_19_N_3_O_8_S	*D*_x_ = 1.521 Mg m^−^^3^
*M**_r_* = 365.36	Mo *K*α radiation, λ = 0.71073 Å
Orthorhombic, *P*2_1_2_1_2_1_	Cell parameters from 9985 reflections
*a* = 7.9857 (4) Å	θ = 3.4–27.5°
*b* = 9.0145 (4) Å	µ = 0.25 mm^−^^1^
*c* = 22.1621 (10) Å	*T* = 90 K
*V* = 1595.39 (13) Å^3^	Cut block, colourless
*Z* = 4	0.30 × 0.28 × 0.20 mm
*F*(000) = 768	

### 2,3:4,5-Bis-O-(1-methylethylidene)-β-D-fructopyranose azidosulfate (I-90K) Data collection

**Table:**

Bruker D8 Venture dual source diffractometer	3662 independent reflections
Radiation source: microsource	3599 reflections with *I* > 2σ(*I*)
Detector resolution: 7.41 pixels mm^-1^	*R*_int_ = 0.031
φ and ω scans	θ_max_ = 27.5°, θ_min_ = 2.4°
Absorption correction: multi-scan *SADABS* (Krause *et al.*, 2015)	*h* = −10→10
*T*_min_ = 0.845, *T*_max_ = 0.958	*k* = −11→11
22942 measured reflections	*l* = −28→28

### 2,3:4,5-Bis-O-(1-methylethylidene)-β-D-fructopyranose azidosulfate (I-90K) Refinement

**Table:**

Refinement on *F*^2^	Secondary atom site location: difference Fourier map
Least-squares matrix: full	Hydrogen site location: difference Fourier map
*R*[*F*^2^ > 2σ(*F*^2^)] = 0.023	H-atom parameters constrained
*wR*(*F*^2^) = 0.060	*w* = 1/[σ^2^(*F*_o_^2^) + (0.0311*P*)^2^ + 0.3897*P*] where *P* = (*F*_o_^2^ + 2*F*_c_^2^)/3
*S* = 1.07	(Δ/σ)_max_ = 0.001
3662 reflections	Δρ_max_ = 0.27 e Å^−^^3^
221 parameters	Δρ_min_ = −0.27 e Å^−^^3^
0 restraints	Absolute structure: Flack *x* determined using 1497 quotients [(*I*^+^)-(*I*^-^)]/[(*I*^+^)+(*I*^-^)] (Parsons *et al.*, 2013)
Primary atom site location: structure-invariant direct methods	Absolute structure parameter: −0.006 (18)

### 2,3:4,5-Bis-O-(1-methylethylidene)-β-D-fructopyranose azidosulfate (I-90K) Special details

**Table:**

Experimental. The crystal was mounted using polyisobutene oil on the tip of a fine glass fibre, which was fastened in a copper mounting pin with electrical solder. It was placed directly into the cold gas stream of a liquid-nitrogen based cryostat (Hope, 1994; Parkin & Hope, 1998). Diffraction data were collected with the crystal at 90K, which is standard practice in this laboratory for the majority of flash-cooled crystals.
Geometry. All esds (except the esd in the dihedral angle between two l.s. planes) are estimated using the full covariance matrix. The cell esds are taken into account individually in the estimation of esds in distances, angles and torsion angles; correlations between esds in cell parameters are only used when they are defined by crystal symmetry. An approximate (isotropic) treatment of cell esds is used for estimating esds involving l.s. planes.
Refinement. Refinement progress was checked using *Platon* (Spek, 2020) and by an *R*-tensor (Parkin, 2000). The final model was further checked with the IUCr utility *checkCIF*.

### 2,3:4,5-Bis-O-(1-methylethylidene)-β-D-fructopyranose azidosulfate (I-90K) Fractional atomic coordinates and isotropic or equivalent isotropic displacement parameters (Å^2^)

**Table:**

	*x*	*y*	*z*	*U*_iso_*/*U*_eq_	
S1	0.74373 (5)	0.79578 (4)	0.39659 (2)	0.01482 (10)	
O1	0.68696 (14)	0.40140 (12)	0.39079 (5)	0.0110 (2)	
O2	0.37338 (14)	0.30579 (13)	0.44523 (5)	0.0132 (2)	
O3	0.29357 (14)	0.40358 (13)	0.35605 (5)	0.0140 (2)	
O4	0.61310 (14)	0.21617 (13)	0.27084 (5)	0.0133 (2)	
O5	0.82007 (14)	0.37553 (13)	0.29850 (5)	0.0129 (2)	
O6	0.79097 (14)	0.66339 (13)	0.35441 (5)	0.0131 (2)	
O7	0.89734 (17)	0.84031 (15)	0.42335 (6)	0.0227 (3)	
O8	0.63978 (18)	0.89829 (14)	0.36559 (6)	0.0233 (3)	
N1	0.61434 (19)	0.72167 (17)	0.44750 (6)	0.0176 (3)	
N2	0.6901 (2)	0.63604 (18)	0.48404 (7)	0.0195 (3)	
N3	0.7424 (3)	0.5607 (2)	0.51895 (7)	0.0303 (4)	
C1	0.6742 (2)	0.42586 (18)	0.32847 (7)	0.0111 (3)	
C2	0.5308 (2)	0.34038 (18)	0.29730 (7)	0.0112 (3)	
H2	0.478553	0.403202	0.265228	0.013*	
C3	0.39740 (19)	0.28231 (19)	0.33997 (7)	0.0121 (3)	
H3	0.329322	0.204201	0.319356	0.015*	
C4	0.46440 (19)	0.22228 (18)	0.40096 (7)	0.0115 (3)	
H4	0.440093	0.113922	0.405033	0.014*	
C5	0.6496 (2)	0.25192 (18)	0.40849 (7)	0.0115 (3)	
H5A	0.682113	0.236671	0.451149	0.014*	
H5B	0.714547	0.181756	0.383316	0.014*	
C6	0.2319 (2)	0.36980 (19)	0.41541 (7)	0.0131 (3)	
C7	0.0877 (2)	0.2609 (2)	0.41185 (7)	0.0172 (4)	
H7A	−0.000562	0.301745	0.386016	0.026*	
H7B	0.042898	0.243492	0.452406	0.026*	
H7C	0.127531	0.166947	0.394831	0.026*	
C8	0.1871 (2)	0.5135 (2)	0.44592 (8)	0.0196 (4)	
H8A	0.285516	0.578470	0.446858	0.029*	
H8B	0.149912	0.493503	0.487260	0.029*	
H8C	0.096764	0.562297	0.423507	0.029*	
C9	0.7769 (2)	0.26520 (18)	0.25392 (7)	0.0132 (3)	
C10	0.8959 (2)	0.1363 (2)	0.25870 (8)	0.0190 (3)	
H10A	0.865031	0.060356	0.229074	0.028*	
H10B	0.889904	0.094120	0.299397	0.028*	
H10C	1.010288	0.170461	0.250730	0.028*	
C11	0.7748 (2)	0.3354 (2)	0.19133 (7)	0.0187 (3)	
H11A	0.748270	0.259460	0.161161	0.028*	
H11B	0.884938	0.378143	0.182579	0.028*	
H11C	0.689687	0.413681	0.190035	0.028*	
C12	0.6546 (2)	0.59209 (18)	0.32148 (7)	0.0130 (3)	
H12A	0.659331	0.619732	0.278277	0.016*	
H12B	0.545271	0.624352	0.337951	0.016*	

### 2,3:4,5-Bis-O-(1-methylethylidene)-β-D-fructopyranose azidosulfate (I-90K) Atomic displacement parameters (Å^2^)

**Table:**

	*U*^11^	*U*^22^	*U*^33^	*U*^12^	*U*^13^	*U*^23^
S1	0.01728 (19)	0.01052 (18)	0.01667 (18)	−0.00154 (16)	0.00150 (16)	−0.00177 (14)
O1	0.0132 (5)	0.0106 (5)	0.0093 (5)	−0.0008 (4)	−0.0014 (4)	0.0007 (4)
O2	0.0101 (5)	0.0190 (6)	0.0104 (5)	0.0033 (5)	−0.0002 (4)	−0.0009 (4)
O3	0.0127 (5)	0.0181 (6)	0.0112 (5)	0.0047 (4)	0.0029 (4)	0.0018 (4)
O4	0.0109 (5)	0.0147 (5)	0.0142 (5)	−0.0016 (5)	0.0031 (4)	−0.0049 (5)
O5	0.0102 (5)	0.0154 (6)	0.0131 (5)	−0.0004 (5)	0.0011 (4)	−0.0052 (4)
O6	0.0126 (5)	0.0120 (5)	0.0148 (5)	−0.0008 (4)	0.0007 (4)	−0.0020 (4)
O7	0.0210 (7)	0.0223 (7)	0.0247 (6)	−0.0071 (6)	0.0008 (5)	−0.0065 (5)
O8	0.0302 (7)	0.0121 (6)	0.0277 (7)	0.0037 (5)	−0.0002 (6)	0.0007 (5)
N1	0.0176 (7)	0.0187 (7)	0.0164 (6)	0.0006 (6)	0.0027 (6)	−0.0004 (6)
N2	0.0209 (7)	0.0195 (7)	0.0180 (7)	−0.0028 (6)	0.0044 (6)	−0.0021 (6)
N3	0.0337 (9)	0.0329 (9)	0.0242 (8)	0.0009 (9)	0.0026 (8)	0.0072 (7)
C1	0.0104 (7)	0.0127 (8)	0.0102 (7)	0.0010 (6)	0.0006 (6)	0.0003 (6)
C2	0.0105 (7)	0.0133 (7)	0.0098 (6)	0.0000 (6)	−0.0005 (6)	−0.0023 (6)
C3	0.0095 (7)	0.0155 (8)	0.0114 (6)	−0.0002 (7)	−0.0003 (6)	−0.0017 (6)
C4	0.0117 (7)	0.0118 (7)	0.0111 (7)	0.0002 (6)	0.0008 (6)	−0.0005 (6)
C5	0.0112 (7)	0.0102 (7)	0.0131 (7)	0.0008 (6)	0.0002 (5)	0.0024 (6)
C6	0.0106 (7)	0.0187 (8)	0.0102 (6)	0.0028 (7)	0.0006 (6)	0.0012 (5)
C7	0.0114 (7)	0.0264 (9)	0.0140 (7)	−0.0016 (7)	0.0000 (6)	0.0015 (7)
C8	0.0210 (8)	0.0187 (9)	0.0190 (8)	0.0051 (7)	0.0052 (7)	−0.0005 (7)
C9	0.0114 (7)	0.0151 (7)	0.0130 (7)	−0.0026 (6)	0.0028 (6)	−0.0042 (6)
C10	0.0159 (8)	0.0192 (8)	0.0217 (8)	0.0028 (7)	0.0026 (7)	−0.0053 (7)
C11	0.0174 (8)	0.0256 (9)	0.0132 (7)	−0.0033 (7)	0.0025 (6)	−0.0014 (6)
C12	0.0138 (8)	0.0122 (7)	0.0130 (7)	−0.0005 (6)	−0.0021 (6)	0.0002 (6)

### 2,3:4,5-Bis-O-(1-methylethylidene)-β-D-fructopyranose azidosulfate (I-90K) Geometric parameters (Å, º)

**Table:**

S1—O8	1.4195 (14)	C4—C5	1.512 (2)
S1—O7	1.4205 (14)	C4—H4	1.0000
S1—O6	1.5622 (12)	C5—H5A	0.9900
S1—N1	1.6694 (15)	C5—H5B	0.9900
O1—C1	1.4022 (18)	C6—C8	1.505 (2)
O1—C5	1.4347 (19)	C6—C7	1.515 (2)
O2—C6	1.4306 (19)	C7—H7A	0.9800
O2—C4	1.4345 (18)	C7—H7B	0.9800
O3—C3	1.4176 (19)	C7—H7C	0.9800
O3—C6	1.4373 (18)	C8—H8A	0.9800
O4—C2	1.4245 (19)	C8—H8B	0.9800
O4—C9	1.4306 (18)	C8—H8C	0.9800
O5—C1	1.4156 (19)	C9—C10	1.505 (2)
O5—C9	1.4436 (19)	C9—C11	1.525 (2)
O6—C12	1.4600 (19)	C10—H10A	0.9800
N1—N2	1.272 (2)	C10—H10B	0.9800
N2—N3	1.111 (2)	C10—H10C	0.9800
C1—C12	1.515 (2)	C11—H11A	0.9800
C1—C2	1.543 (2)	C11—H11B	0.9800
C2—C3	1.518 (2)	C11—H11C	0.9800
C2—H2	1.0000	C12—H12A	0.9900
C3—C4	1.551 (2)	C12—H12B	0.9900
C3—H3	1.0000		
			
O8—S1—O7	121.55 (8)	H5A—C5—H5B	108.2
O8—S1—O6	110.42 (7)	O2—C6—O3	103.74 (12)
O7—S1—O6	104.91 (7)	O2—C6—C8	109.11 (13)
O8—S1—N1	103.04 (8)	O3—C6—C8	108.07 (14)
O7—S1—N1	111.43 (8)	O2—C6—C7	111.28 (13)
O6—S1—N1	104.37 (7)	O3—C6—C7	110.49 (13)
C1—O1—C5	113.70 (12)	C8—C6—C7	113.64 (14)
C6—O2—C4	107.21 (11)	C6—C7—H7A	109.5
C3—O3—C6	105.50 (12)	C6—C7—H7B	109.5
C2—O4—C9	106.66 (12)	H7A—C7—H7B	109.5
C1—O5—C9	110.20 (12)	C6—C7—H7C	109.5
C12—O6—S1	117.09 (10)	H7A—C7—H7C	109.5
N2—N1—S1	112.27 (12)	H7B—C7—H7C	109.5
N3—N2—N1	173.36 (19)	C6—C8—H8A	109.5
O1—C1—O5	110.61 (13)	C6—C8—H8B	109.5
O1—C1—C12	105.31 (13)	H8A—C8—H8B	109.5
O5—C1—C12	110.74 (13)	C6—C8—H8C	109.5
O1—C1—C2	114.60 (13)	H8A—C8—H8C	109.5
O5—C1—C2	103.91 (12)	H8B—C8—H8C	109.5
C12—C1—C2	111.80 (13)	O4—C9—O5	104.59 (11)
O4—C2—C3	108.01 (13)	O4—C9—C10	108.69 (14)
O4—C2—C1	103.56 (12)	O5—C9—C10	109.45 (13)
C3—C2—C1	114.46 (13)	O4—C9—C11	110.88 (13)
O4—C2—H2	110.2	O5—C9—C11	109.83 (13)
C3—C2—H2	110.2	C10—C9—C11	113.04 (14)
C1—C2—H2	110.2	C9—C10—H10A	109.5
O3—C3—C2	107.54 (13)	C9—C10—H10B	109.5
O3—C3—C4	104.57 (12)	H10A—C10—H10B	109.5
C2—C3—C4	114.90 (13)	C9—C10—H10C	109.5
O3—C3—H3	109.9	H10A—C10—H10C	109.5
C2—C3—H3	109.9	H10B—C10—H10C	109.5
C4—C3—H3	109.9	C9—C11—H11A	109.5
O2—C4—C5	109.11 (13)	C9—C11—H11B	109.5
O2—C4—C3	103.78 (12)	H11A—C11—H11B	109.5
C5—C4—C3	111.83 (13)	C9—C11—H11C	109.5
O2—C4—H4	110.6	H11A—C11—H11C	109.5
C5—C4—H4	110.6	H11B—C11—H11C	109.5
C3—C4—H4	110.6	O6—C12—C1	107.89 (13)
O1—C5—C4	109.82 (13)	O6—C12—H12A	110.1
O1—C5—H5A	109.7	C1—C12—H12A	110.1
C4—C5—H5A	109.7	O6—C12—H12B	110.1
O1—C5—H5B	109.7	C1—C12—H12B	110.1
C4—C5—H5B	109.7	H12A—C12—H12B	108.4
			
O8—S1—O6—C12	−48.73 (13)	C6—O2—C4—C5	136.56 (13)
O7—S1—O6—C12	178.68 (11)	C6—O2—C4—C3	17.21 (15)
N1—S1—O6—C12	61.39 (12)	O3—C3—C4—O2	6.99 (15)
O8—S1—N1—N2	−173.57 (13)	C2—C3—C4—O2	124.63 (14)
O7—S1—N1—N2	−41.67 (15)	O3—C3—C4—C5	−110.49 (14)
O6—S1—N1—N2	71.03 (13)	C2—C3—C4—C5	7.15 (19)
C5—O1—C1—O5	−80.38 (16)	C1—O1—C5—C4	−70.46 (16)
C5—O1—C1—C12	159.94 (12)	O2—C4—C5—O1	−69.49 (16)
C5—O1—C1—C2	36.65 (18)	C3—C4—C5—O1	44.74 (17)
C9—O5—C1—O1	121.68 (13)	C4—O2—C6—O3	−35.14 (15)
C9—O5—C1—C12	−121.96 (14)	C4—O2—C6—C8	−150.14 (14)
C9—O5—C1—C2	−1.77 (16)	C4—O2—C6—C7	83.68 (15)
C9—O4—C2—C3	−154.60 (12)	C3—O3—C6—O2	39.69 (15)
C9—O4—C2—C1	−32.84 (15)	C3—O3—C6—C8	155.43 (13)
O1—C1—C2—O4	−99.81 (14)	C3—O3—C6—C7	−79.67 (16)
O5—C1—C2—O4	21.00 (15)	C2—O4—C9—O5	32.11 (15)
C12—C1—C2—O4	140.47 (13)	C2—O4—C9—C10	148.94 (13)
O1—C1—C2—C3	17.5 (2)	C2—O4—C9—C11	−86.21 (15)
O5—C1—C2—C3	138.33 (14)	C1—O5—C9—O4	−18.10 (16)
C12—C1—C2—C3	−102.20 (16)	C1—O5—C9—C10	−134.40 (14)
C6—O3—C3—C2	−150.97 (12)	C1—O5—C9—C11	100.94 (15)
C6—O3—C3—C4	−28.39 (15)	S1—O6—C12—C1	−133.17 (11)
O4—C2—C3—O3	−167.78 (12)	O1—C1—C12—O6	52.54 (16)
C1—C2—C3—O3	77.46 (16)	O5—C1—C12—O6	−67.06 (16)
O4—C2—C3—C4	76.26 (16)	C2—C1—C12—O6	177.58 (12)
C1—C2—C3—C4	−38.49 (19)		

### 2,3:4,5-Bis-O-(1-methylethylidene)-β-D-fructopyranose azidosulfate (I-90K) Hydrogen-bond geometry (Å, º)

**Table:**

*D*—H···*A*	*D*—H	H···*A*	*D*···*A*	*D*—H···*A*
C7—H7*A*···O1^i^	0.98	2.65	3.473 (2)	141
C7—H7*A*···O5^i^	0.98	2.50	3.456 (2)	165
C5—H5*B*···O8^ii^	0.99	2.65	3.328 (2)	126
C12—H12*A*···O4^iii^	0.99	2.58	3.163 (2)	117

Symmetry codes: (i) *x*−1, *y*, *z*; (ii) *x*, *y*−1, *z*; (iii) −*x*+1, *y*+1/2, −*z*+1/2.

### (I-298K) Crystal data

**Table:**

C_12_H_19_N_3_O_8_S	*D*_x_ = 1.466 Mg m^−^^3^
*M**_r_* = 365.36	Mo *K*α radiation, λ = 0.71073 Å
Orthorhombic, *P*2_1_2_1_2_1_	Cell parameters from 9504 reflections
*a* = 8.0717 (8) Å	θ = 3.4–27.5°
*b* = 9.1135 (12) Å	µ = 0.24 mm^−^^1^
*c* = 22.506 (3) Å	*T* = 298 K
*V* = 1655.6 (3) Å^3^	Cut block, colourless
*Z* = 4	0.24 × 0.22 × 0.14 mm
*F*(000) = 768	

### (I-298K) Data collection

**Table:**

Bruker D8 Venture dual source diffractometer	3786 independent reflections
Radiation source: microsource	3523 reflections with *I* > 2σ(*I*)
Detector resolution: 7.41 pixels mm^-1^	*R*_int_ = 0.067
φ and ω scans	θ_max_ = 27.5°, θ_min_ = 2.4°
Absorption correction: multi-scan (*SADABS*; Krause *et al.*, 2015)	*h* = −10→10
*T*_min_ = 0.815, *T*_max_ = 0.959	*k* = −11→11
22913 measured reflections	*l* = −29→25

### (I-298K) Refinement

**Table:**

Refinement on *F*^2^	Secondary atom site location: difference Fourier map
Least-squares matrix: full	Hydrogen site location: difference Fourier map
*R*[*F*^2^ > 2σ(*F*^2^)] = 0.036	H-atom parameters constrained
*wR*(*F*^2^) = 0.099	*w* = 1/[σ^2^(*F*_o_^2^) + (0.0574*P*)^2^ + 0.1732*P*] where *P* = (*F*_o_^2^ + 2*F*_c_^2^)/3
*S* = 1.04	(Δ/σ)_max_ < 0.001
3786 reflections	Δρ_max_ = 0.20 e Å^−^^3^
221 parameters	Δρ_min_ = −0.27 e Å^−^^3^
0 restraints	Absolute structure: Flack *x* determined using 1388 quotients [(*I*^+^)-(*I*^-^)]/[(*I*^+^)+(*I*^-^)] (Parsons *et al.*, 2013)
Primary atom site location: structure-invariant direct methods	Absolute structure parameter: 0.07 (5)

### (I-298K) Special details

**Table:**

Experimental. The crystal was mounted glued to the tip of a fine glass fibre, which was fastened in a copper mounting pin with electrical solder. Data were collected at room temperature to investigate the possibility of the methyl group at C7 undergoing rapid spinning.
Geometry. All esds (except the esd in the dihedral angle between two l.s. planes) are estimated using the full covariance matrix. The cell esds are taken into account individually in the estimation of esds in distances, angles and torsion angles; correlations between esds in cell parameters are only used when they are defined by crystal symmetry. An approximate (isotropic) treatment of cell esds is used for estimating esds involving l.s. planes.
Refinement. Refinement progress was checked using *Platon* (Spek, 2020) and by an *R*-tensor (Parkin, 2000). The final model was further checked with the IUCr utility *checkCIF*.

### (I-298K) Fractional atomic coordinates and isotropic or equivalent isotropic displacement parameters (Å^2^)

**Table:**

	*x*	*y*	*z*	*U*_iso_*/*U*_eq_	
S1	0.74935 (9)	0.79040 (6)	0.39549 (3)	0.05008 (19)	
O1	0.68675 (18)	0.39774 (16)	0.38959 (6)	0.0334 (3)	
O2	0.3760 (2)	0.3015 (2)	0.44310 (7)	0.0418 (4)	
O3	0.29761 (19)	0.4019 (2)	0.35639 (7)	0.0444 (4)	
O4	0.6115 (2)	0.22006 (19)	0.27062 (7)	0.0405 (4)	
O5	0.81632 (19)	0.37532 (19)	0.29858 (8)	0.0406 (4)	
O6	0.7900 (2)	0.65798 (18)	0.35461 (8)	0.0424 (4)	
O7	0.9030 (3)	0.8324 (3)	0.42018 (11)	0.0739 (7)	
O8	0.6469 (4)	0.8910 (2)	0.36562 (13)	0.0810 (7)	
N1	0.6247 (3)	0.7208 (3)	0.44695 (12)	0.0586 (6)	
N2	0.6998 (4)	0.6366 (3)	0.48301 (13)	0.0658 (7)	
N3	0.7518 (6)	0.5639 (5)	0.51657 (17)	0.1031 (12)	
C1	0.6730 (3)	0.4242 (2)	0.32874 (9)	0.0317 (4)	
C2	0.5312 (3)	0.3413 (3)	0.29776 (9)	0.0345 (4)	
H2	0.479985	0.403642	0.267422	0.041*	
C3	0.3998 (2)	0.2823 (3)	0.33972 (9)	0.0356 (4)	
H3	0.333367	0.207389	0.319499	0.043*	
C4	0.4662 (3)	0.2211 (2)	0.39892 (10)	0.0349 (4)	
H4	0.442711	0.115874	0.402132	0.042*	
C5	0.6487 (3)	0.2499 (2)	0.40661 (10)	0.0354 (5)	
H5A	0.679702	0.234428	0.447786	0.042*	
H5B	0.711710	0.181983	0.382284	0.042*	
C6	0.2387 (3)	0.3687 (3)	0.41482 (10)	0.0414 (5)	
C7	0.0929 (3)	0.2650 (4)	0.41220 (13)	0.0581 (8)	
H7A	0.005298	0.309049	0.389497	0.087*	
H7B	0.054350	0.245317	0.451757	0.087*	
H7C	0.126270	0.174873	0.393681	0.087*	
C8	0.2014 (4)	0.5115 (4)	0.44567 (16)	0.0670 (9)	
H8A	0.299085	0.571603	0.446096	0.101*	
H8B	0.166869	0.492278	0.485738	0.101*	
H8C	0.114454	0.561748	0.424853	0.101*	
C9	0.7734 (3)	0.2684 (3)	0.25411 (10)	0.0420 (5)	
C10	0.8905 (4)	0.1406 (3)	0.25816 (15)	0.0605 (7)	
H10A	0.860101	0.067868	0.229299	0.091*	
H10B	0.884762	0.098732	0.297250	0.091*	
H10C	1.001458	0.173537	0.250531	0.091*	
C11	0.7710 (4)	0.3396 (4)	0.19315 (12)	0.0612 (7)	
H11A	0.742453	0.267603	0.163763	0.092*	
H11B	0.878574	0.379166	0.184444	0.092*	
H11C	0.690558	0.417191	0.192682	0.092*	
C12	0.6536 (3)	0.5884 (2)	0.32299 (11)	0.0399 (5)	
H12A	0.655642	0.616531	0.281430	0.048*	
H12B	0.548641	0.619096	0.339866	0.048*	

### (I-298K) Atomic displacement parameters (Å^2^)

**Table:**

	*U*^11^	*U*^22^	*U*^33^	*U*^12^	*U*^13^	*U*^23^
S1	0.0602 (4)	0.0303 (3)	0.0598 (4)	−0.0049 (3)	0.0056 (3)	−0.0074 (2)
O1	0.0365 (7)	0.0311 (7)	0.0326 (7)	−0.0014 (6)	−0.0052 (6)	0.0011 (6)
O2	0.0355 (7)	0.0565 (10)	0.0333 (7)	0.0088 (8)	−0.0010 (6)	−0.0008 (7)
O3	0.0373 (8)	0.058 (1)	0.0377 (8)	0.0136 (7)	0.0045 (7)	0.0071 (7)
O4	0.0382 (7)	0.0418 (8)	0.0416 (8)	−0.0060 (7)	0.0071 (7)	−0.0148 (7)
O5	0.0312 (7)	0.0451 (9)	0.0455 (9)	−0.0025 (7)	0.0042 (6)	−0.0133 (7)
O6	0.0441 (9)	0.0327 (8)	0.0505 (9)	−0.0036 (6)	0.0048 (7)	−0.0046 (7)
O7	0.0710 (14)	0.0659 (14)	0.0848 (16)	−0.0266 (12)	0.0040 (12)	−0.0256 (12)
O8	0.110 (2)	0.0347 (10)	0.0983 (17)	0.0140 (12)	−0.0007 (16)	0.0037 (11)
N1	0.0585 (13)	0.0592 (14)	0.0583 (13)	0.0015 (12)	0.0130 (11)	−0.0079 (12)
N2	0.0774 (18)	0.0630 (16)	0.0570 (15)	−0.0066 (14)	0.0089 (14)	−0.0031 (13)
N3	0.127 (3)	0.105 (3)	0.077 (2)	0.003 (3)	0.000 (3)	0.026 (2)
C1	0.0312 (9)	0.0324 (10)	0.0315 (10)	0.0009 (8)	−0.0007 (8)	−0.0017 (8)
C2	0.0323 (10)	0.0420 (11)	0.0292 (10)	0.0006 (8)	−0.0005 (8)	−0.0045 (9)
C3	0.0276 (8)	0.0439 (11)	0.0353 (10)	−0.0019 (9)	−0.0002 (8)	−0.0058 (9)
C4	0.0332 (9)	0.0333 (10)	0.0382 (11)	0.0012 (8)	0.0028 (8)	0.0009 (9)
C5	0.0338 (10)	0.0305 (10)	0.0417 (11)	0.0041 (8)	−0.0017 (8)	0.0061 (8)
C6	0.0343 (10)	0.0544 (13)	0.0354 (11)	0.0097 (11)	0.0028 (9)	0.0025 (9)
C7	0.0346 (11)	0.091 (2)	0.0489 (14)	−0.0055 (13)	0.0014 (10)	0.0063 (15)
C8	0.075 (2)	0.0620 (18)	0.0640 (18)	0.0221 (16)	0.0206 (16)	−0.0047 (15)
C9	0.0373 (11)	0.0472 (12)	0.0416 (11)	−0.0022 (10)	0.0072 (9)	−0.013 (1)
C10	0.0526 (15)	0.0566 (15)	0.0722 (18)	0.0116 (14)	0.0114 (14)	−0.0184 (14)
C11	0.0600 (16)	0.0788 (19)	0.0449 (14)	−0.0095 (16)	0.0106 (13)	−0.0052 (13)
C12	0.0450 (12)	0.0331 (11)	0.0415 (12)	0.0021 (9)	−0.0049 (9)	0.0020 (9)

### (I-298K) Geometric parameters (Å, º)

**Table:**

S1—O8	1.406 (3)	C4—C5	1.507 (3)
S1—O7	1.412 (2)	C4—H4	0.9800
S1—O6	1.5527 (17)	C5—H5A	0.9700
S1—N1	1.660 (3)	C5—H5B	0.9700
O1—C1	1.395 (3)	C6—C8	1.506 (4)
O1—C5	1.434 (3)	C6—C7	1.511 (4)
O2—C6	1.417 (3)	C7—H7A	0.9600
O2—C4	1.434 (3)	C7—H7B	0.9600
O3—C3	1.418 (3)	C7—H7C	0.9600
O3—C6	1.431 (3)	C8—H8A	0.9600
O4—C2	1.419 (3)	C8—H8B	0.9600
O4—C9	1.429 (3)	C8—H8C	0.9600
O5—C1	1.413 (3)	C9—C10	1.503 (4)
O5—C9	1.439 (3)	C9—C11	1.518 (4)
O6—C12	1.457 (3)	C10—H10A	0.9600
N1—N2	1.271 (4)	C10—H10B	0.9600
N2—N3	1.089 (5)	C10—H10C	0.9600
C1—C12	1.510 (3)	C11—H11A	0.9600
C1—C2	1.539 (3)	C11—H11B	0.9600
C2—C3	1.518 (3)	C11—H11C	0.9600
C2—H2	0.9800	C12—H12A	0.9700
C3—C4	1.540 (3)	C12—H12B	0.9700
C3—H3	0.9800		
			
O8—S1—O7	121.86 (16)	H5A—C5—H5B	108.2
O8—S1—O6	110.36 (14)	O2—C6—O3	104.16 (17)
O7—S1—O6	104.96 (12)	O2—C6—C8	108.8 (2)
O8—S1—N1	103.09 (17)	O3—C6—C8	107.9 (2)
O7—S1—N1	111.19 (15)	O2—C6—C7	110.9 (2)
O6—S1—N1	104.17 (11)	O3—C6—C7	110.8 (2)
C1—O1—C5	114.07 (16)	C8—C6—C7	113.7 (2)
C6—O2—C4	107.84 (16)	C6—C7—H7A	109.5
C3—O3—C6	105.87 (17)	C6—C7—H7B	109.5
C2—O4—C9	106.84 (16)	H7A—C7—H7B	109.5
C1—O5—C9	110.53 (16)	C6—C7—H7C	109.5
C12—O6—S1	117.92 (14)	H7A—C7—H7C	109.5
N2—N1—S1	112.8 (2)	H7B—C7—H7C	109.5
N3—N2—N1	173.9 (4)	C6—C8—H8A	109.5
O1—C1—O5	110.61 (17)	C6—C8—H8B	109.5
O1—C1—C12	105.30 (17)	H8A—C8—H8B	109.5
O5—C1—C12	110.87 (19)	C6—C8—H8C	109.5
O1—C1—C2	114.75 (18)	H8A—C8—H8C	109.5
O5—C1—C2	103.67 (16)	H8B—C8—H8C	109.5
C12—C1—C2	111.76 (18)	O4—C9—O5	104.36 (16)
O4—C2—C3	108.12 (18)	O4—C9—C10	108.7 (2)
O4—C2—C1	103.75 (16)	O5—C9—C10	109.3 (2)
C3—C2—C1	114.32 (17)	O4—C9—C11	110.8 (2)
O4—C2—H2	110.1	O5—C9—C11	110.0 (2)
C3—C2—H2	110.1	C10—C9—C11	113.2 (2)
C1—C2—H2	110.1	C9—C10—H10A	109.5
O3—C3—C2	107.36 (18)	C9—C10—H10B	109.5
O3—C3—C4	104.59 (17)	H10A—C10—H10B	109.5
C2—C3—C4	115.04 (17)	C9—C10—H10C	109.5
O3—C3—H3	109.9	H10A—C10—H10C	109.5
C2—C3—H3	109.9	H10B—C10—H10C	109.5
C4—C3—H3	109.9	C9—C11—H11A	109.5
O2—C4—C5	109.14 (18)	C9—C11—H11B	109.5
O2—C4—C3	103.79 (16)	H11A—C11—H11B	109.5
C5—C4—C3	112.11 (18)	C9—C11—H11C	109.5
O2—C4—H4	110.5	H11A—C11—H11C	109.5
C5—C4—H4	110.5	H11B—C11—H11C	109.5
C3—C4—H4	110.5	O6—C12—C1	108.13 (18)
O1—C5—C4	110.00 (17)	O6—C12—H12A	110.1
O1—C5—H5A	109.7	C1—C12—H12A	110.1
C4—C5—H5A	109.7	O6—C12—H12B	110.1
O1—C5—H5B	109.7	C1—C12—H12B	110.1
C4—C5—H5B	109.7	H12A—C12—H12B	108.4
			
O8—S1—O6—C12	−47.7 (2)	C6—O2—C4—C5	135.43 (19)
O7—S1—O6—C12	179.27 (18)	C6—O2—C4—C3	15.7 (2)
N1—S1—O6—C12	62.31 (19)	O3—C3—C4—O2	7.4 (2)
O8—S1—N1—N2	−173.4 (2)	C2—C3—C4—O2	124.95 (19)
O7—S1—N1—N2	−41.2 (3)	O3—C3—C4—C5	−110.21 (19)
O6—S1—N1—N2	71.3 (2)	C2—C3—C4—C5	7.3 (3)
C5—O1—C1—O5	−80.8 (2)	C1—O1—C5—C4	−69.5 (2)
C5—O1—C1—C12	159.36 (17)	O2—C4—C5—O1	−70.3 (2)
C5—O1—C1—C2	36.0 (2)	C3—C4—C5—O1	44.1 (2)
C9—O5—C1—O1	121.76 (19)	C4—O2—C6—O3	−33.2 (2)
C9—O5—C1—C12	−121.8 (2)	C4—O2—C6—C8	−148.0 (2)
C9—O5—C1—C2	−1.7 (2)	C4—O2—C6—C7	86.1 (2)
C9—O4—C2—C3	−154.34 (17)	C3—O3—C6—O2	38.0 (2)
C9—O4—C2—C1	−32.6 (2)	C3—O3—C6—C8	153.5 (2)
O1—C1—C2—O4	−99.94 (19)	C3—O3—C6—C7	−81.4 (2)
O5—C1—C2—O4	20.8 (2)	C2—O4—C9—O5	31.7 (2)
C12—C1—C2—O4	140.27 (19)	C2—O4—C9—C10	148.3 (2)
O1—C1—C2—C3	17.6 (3)	C2—O4—C9—C11	−86.6 (2)
O5—C1—C2—C3	138.32 (19)	C1—O5—C9—O4	−17.9 (2)
C12—C1—C2—C3	−102.2 (2)	C1—O5—C9—C10	−134.0 (2)
C6—O3—C3—C2	−150.25 (18)	C1—O5—C9—C11	101.1 (2)
C6—O3—C3—C4	−27.6 (2)	S1—O6—C12—C1	−135.17 (17)
O4—C2—C3—O3	−167.48 (17)	O1—C1—C12—O6	53.4 (2)
C1—C2—C3—O3	77.5 (2)	O5—C1—C12—O6	−66.3 (2)
O4—C2—C3—C4	76.6 (2)	C2—C1—C12—O6	178.62 (17)
C1—C2—C3—C4	−38.4 (3)		

### (I-298K) Hydrogen-bond geometry (Å, º)

**Table:**

*D*—H···*A*	*D*—H	H···*A*	*D*···*A*	*D*—H···*A*
C7—H7*A*···O1^i^	0.96	2.70	3.531 (3)	146
C7—H7*A*···O5^i^	0.96	2.62	3.540 (3)	160
C5—H5*B*···O8^ii^	0.97	2.73	3.398 (3)	127
C12—H12*A*···O4^iii^	0.97	2.63	3.234 (3)	121

Symmetry codes: (i) *x*−1, *y*, *z*; (ii) *x*, *y*−1, *z*; (iii) −*x*+1, *y*+1/2, −*z*+1/2.
